# An Unusual Cause of Acute Right-Sided Heart Failure Presenting With Refractory Hypoxia

**DOI:** 10.7759/cureus.40872

**Published:** 2023-06-23

**Authors:** Ifeoma Kwentoh, Terrence Henry, Padmaja Sajja

**Affiliations:** 1 Medicine, Columbia University, New York, USA; 2 Internal Medicine, Harlem Hospital Center, New York, USA; 3 Pulmonary and Critical Care Medicine, Harlem Hospital Center, New York, USA

**Keywords:** pocus (point of care ultrasound), interatrial septal aneurysm, pulmonary hypertension, aneurysmal interatrial septum, tricuspid regurgitation, intracardiac shunt, patent forman ovale (pfo)

## Abstract

Atrial septal aneurysm (ASA) formation is due to a deformity at the fossa ovalis. While previously considered a rare cardiac anomaly found postmortem, it can now be diagnosed at the bedside with ultrasound. Unrepaired ASA can lead to right-sided heart failure and pulmonary hypertension. The case we describe is complicated by the patient’s code status, limiting our ability to perform potential life-sustaining interventions. We also encountered a complication of rebound pulmonary hypertension with the use of inhaled nitric oxide. We detail the critical course of profound hemodynamic and respiratory instability responsive to salvage therapy.

## Introduction

Patients requiring critical care admission may present with confounding signs indicating compromise of multiple organ systems, making it difficult to establish a diagnosis. We report a case of an elderly male presenting with acute hypoxemic respiratory failure initially considered secondary to pulmonary compromise, for which chest X-ray findings were suggestive of an infectious process but ultimately found to have an atrial intracardiac shunt leading to severe decompensated right-sided heart failure [1].

## Case presentation

A 71-year-old male with a prior history of chronic obstructive pulmonary disease (COPD Group E) on 4 L of home oxygen and HIV was brought in by EMS with complaints of dyspnea, leg swelling, and hypoxia to ’60s with a home pulse oximeter for five days prior to his presentation. He was ill-appearing with a blood pressure of 121/97 and was hypoxic with an SPO_2_ of 88% on room air. Physical examination showed diffuse bilateral end-expiratory wheezing, pitting edema extending to his thighs, and cool lower extremities. Labs showed acute kidney injury with creatinine of 2.1 and high anion gap metabolic acidosis with lactic acidosis (pH 7.22, pCO_2_ 43, lactate 8.1, HCO_3_ 19, and anion gap 23). A chest X-ray showed mild pulmonary vascular congestion with trace pleural effusions and cardiomegaly suggestive of vascular congestion and infectious process. Electrocardiogram (EKG) showed sinus rhythm with premature atrial contractions and right bundle branch block similar to previous findings. 

Standard therapy with intravenous corticosteroids, antibiotics, and nebulization was initiated for COPD exacerbation and possible pneumonia. Acute pulmonary embolism and decompensated heart failure were entertained differentials. The patient was placed on noninvasive positive pressure ventilation (bilevel positive airway pressure (BIPAP)) and admitted to the intensive care unit (ICU). During his early ICU course, the patient remained persistently hypoxic, developed acute encephalopathy, was unable to maintain his airway, and was intubated. The arterial-alveolar gradient was wide, and maximal ventilatory support (FiO_2_ 80%, tidal volume (VT) 450, respiratory rate (RR) 24, positive end-expiratory pressure (PEEP) 12) was needed to maintain oxygenation. Vasopressor support with norepinephrine was initiated for presumed cardiogenic shock.

Pulmonary embolism was ruled out by a CT pulmonary angiogram, and intravenous diuresis with furosemide was initiated to optimize volume status. A bedside point-of-care ultrasound (POCUS) showed a dilated right ventricle (RV), signs of pressure, and volume overload with a possible intracardiac atrial shunt. Intracardiac shunt was confirmed on a transthoracic echocardiogram (TTE) with a bubble study, which showed hyperdynamic left ventricular systolic function, dilated left and right atrium, severe tricuspid regurgitation with pulmonary hypertension, and intracardiac shunt with a notable aneurysmal interatrial septum, a new incidental finding when compared to prior echocardiograms (Video 1). Inhaled nitric oxide (NO) was initiated at 10 ppm to assist in the dilation of the pulmonary vasculature in order to improve oxygenation. With continued diuresis and NO treatment, oxygenation improved; however, hypoxemia persisted and was refractory to NO titration. Unfortunately, no further invasive diagnostic modalities were pursued post-intubation, as the patient’s family requested Do Not Resuscitate (DNR) status with the continuation of current life-sustaining measures only. He ultimately demised.

**Video 1:** 2D echocardiogram: bubble study with agitated saline. Bubble study shows bulging of ASA on the administration of saline flush. ASA: atrial septal aneurysm.

## Discussion

Intracardiac shunts are anomalous pathways for blood flow within cardiac chambers that are formed either in addition to or in place of normal pathways for blood flow. Atrial septum aneurysm (ASA) is a rare cardiac abnormality described as a localized deformity at the level of the fossa ovalis, which protrudes to the right or left atrium or on both sides [2]. ASA formation is linked to an interatrial pressure difference or primary malformation involving the fossa ovalis. Its presence leads to left-to-right shunts in about 70% of patients diagnosed with the deformity. A non-ejection systolic click is occasionally heard on examination as the interatrial septal aneurysm bulges and tenses within the left atrial (LA)/right atrial (RA) cavity. Shunting over time leads to the development of pulmonary arterial hypertension. Failure to repair the lesion early may lead to persistent exposure of the pulmonary vasculature to increased blood flow and pressure, resulting in vascular remodeling and dysfunction.

Pulmonary hypertension ensues, causing chronically elevated right ventricular afterload and progressive RV dysfunction, which can precipitate a sudden deterioration in right ventricular function [3]. Our patient likely had a congenital patent fossa ovalis, albeit small, which over time led to the formation of the ASA that went undiagnosed for years. The gradual development of increased RA pressures and his history of COPD would have played a part in the development of pulmonary hypertension, a double insult. Our patient developed acute respiratory failure (Eisenmenger syndrome) from decompensated right-sided heart failure complicated by possible pneumonia. Given the limitations within the context of the patient’s code status in performing further invasive studies and interventions, NO therapy was initiated at 10 ppm and then up-titrated to 20 ppm to improve right ventricular function by selective pulmonary vasodilation, which in turn improved left ventricular filling, cardiac output, and systemic arterial pressure. With this, we were able to successfully wean vasopressor support, which was transiently started for cardiogenic shock.

Hemodynamics remained stable, and aggressive intravenous diuresis with furosemide proved beneficial in maintaining a negative fluid balance and improving his renal function. However, we developed a challenge, though NO had successfully improved cardiac output and hypoxemia, with weaning attempts, the patient became tachycardic with bouts of paroxysmal atrial fibrillation, hypoxia, and hypoxemia. Intraventricular dependency appeared unchanged throughout this time, and chest imaging showed no worsening features. We suspected this could be related to rebound pulmonary hypertension, a potentially deadly side effect of inhaled vasodilator withdrawal. Rebound pulmonary hypertension is characterized by increased pulmonary vascular resistance, impaired cardiac function, and severe hypoxemia [4]. With very small incremental daily titration, we were eventually able to wean the patient off NO therapy and perform successful extubation. This method of inhaled NO is considered salvage therapy for refractory hypoxia, as the effects of NO are usually short-lived and demonstrate no mortality benefit with continued use. The patient's respiratory status remained stable following the discontinuation of NO, without requiring any additional therapy. He, however, demised two days post-discontinuation of NO therapy. 

## Conclusions

Patients with intracardiac shunts will develop pulmonary hypertension if the primary etiology of the shunt formation is not addressed early. Inhaled vasodilatory therapy may prove beneficial in the management of patients presenting with decompensated right-sided heart failure. One must be cognizant of the fact that NO therapy, if withdrawn quickly, may precipitate rebound pulmonary hypertension, which can compromise previously stable hemodynamics.
